# Pumpless microfluidic system driven by hydrostatic pressure induces and maintains mouse spermatogenesis *in vitro*

**DOI:** 10.1038/s41598-017-15799-3

**Published:** 2017-11-13

**Authors:** Mitsuru Komeya, Kazuaki Hayashi, Hiroko Nakamura, Hiroyuki Yamanaka, Hiroyuki Sanjo, Kazuaki Kojima, Takuya Sato, Masahiro Yao, Hiroshi Kimura, Teruo Fujii, Takehiko Ogawa

**Affiliations:** 1 Laboratory of Biopharmaceutical and Regenerative Sciences, Institute of Molecular Medicine and Life Science, Yokohama City University Association of Medical Science, Yokohama, Kanagawa 236-0004 Japan; 2 Department of Urology, Yokohama City University Graduate School of Medicine, Yokohama, Kanagawa 236-0004 Japan; 3 Department of Mechanical Engineering, Tokai University, Hiratsuka, Kanagawa 259-1292 Japan; 4 Institute of Industrial Science, University of Tokyo, Komaba, Meguro-ku, Tokyo, 153-8505 Japan

## Abstract

Three-dimensional aggregation and organ culture methods are critical for recreating *in vivo* cellular phenomena outside the body. Previously, we used the conventional gas liquid interphase organ culture method to induce complete mouse spermatogenesis. After incorporating microfluidic systems, we achieved a significant increase in efficiency and duration of spermatogenesis. One of the major drawbacks preventing the popularization of microfluidics, however, is the use of a power-pump to generate medium flow. In this study, we produced a pumpless microfluidic device using hydrostatic pressure and a resistance circuit to facilitate slow, longer lasting medium flow. During three months of culture, results in induction and maintenance of spermatogenesis showed no difference between pumpless and pump-driven devices. Correspondingly, the spermatogonial population was favorably maintained in the pumpless device compared to the conventional method. These results show the advantage of using microfluidic systems for organ culture experiments. Our pumpless device could be applied to a variety of other tissues and organs, and may revolutionize organ culture methods as a whole.

## Introduction

Spermatogenesis is a complex process of cell proliferation and differentiation, which is difficult to recapitulate *ex vivo*. In 2011, we cultured mouse testis tissues with the classical organ culture method that depends on the air-liquid interphase principle, and succeeded in the production of fertile sperm from spermatogonial stem cells^1,2^. However, the efficiency and duration of *in vitro* spermatogenesis were low and limited, respectively, being totally incomparable with those *in vivo*. In order to improve the culture system, we introduced microfluidic technology derived from a technique to manufacture semiconductors. In our microfluidic device, the culture medium flowed from the reservoir tank to the exit through a narrow channel. The testis tissue was spread in a shallow chamber of the device separated by a porous membrane from the flowing medium. Molecules of both nutritionals and waste-products diffused between the chamber and channel through the porous membrane to support the metabolism of the tissue. Oxygen could reach the tissue through the substrate of the device, polydimethylsiloxane (PDMS), which is highly oxygen-permeable. This microfluidic device maintained the two essential functions of the testis, spermatogenesis and testosterone production, for more than 6 months^3^. In addition, the device provided higher visibility of the tissue in culture, which even allows the identification of meiotic and haploid cells when GFP markers are incorporated. Thus, it enables us to examine the details of spermatogenic progression at cellular and even sub-cellular levels.

The microfluidics system is promising for improving organ culture experiments, involving not only testis tissue but possibly other tissues and organs as well. However, there are several limitations which impede its popularization. One of them is the external syringe pump for the flow of medium. Simply put, the number of pumps limits the number of samples studied. The tube that connects the device and pump could be problematic, especially when the sample number is increased. One way to circumvent this is to use a micro-pump integrated into the device^4–6^. However, such pump-integrated devices require expert- fabrication and are expensive. Consequently, a pump-free device has been demanded and various prototypes have been produced^7–20^. They are divided into four categories based on their flow-actuating mechanisms^21^; gravity-driven^7–15^, surface tension-driven^16,17^, osmosis-driven^18,19^, and others depending on external-power sources^20^. Some devices were successfully refined to improve their performance for maintaining a constant flow^7,10,12,13,15^. Until the present, however, these devices have been applied exclusively for cell culture experiments on a small scale and relatively for short periods of time. Only one exceptional trial, to our knowledge, reported that a skin organoid was maintained for three weeks in a device. This device was pump-free but used a rocking platform, which is thus external power-dependent^20^. In the present study, we utilized the hydrostatic pressure to induce the flow of culture medium in a device free from a power-driven pump altogether. This pumpless (PL) device successfully induced mouse spermatogenesis from primitive spermatogonia up to haploid cell formation and maintained it for 3 months. This is the first report of a power-free perfusion system in a microfluidic device maintaining the function and architecture of testis tissue for long periods.

## Results

### Design of PL device

**Figure 1:**
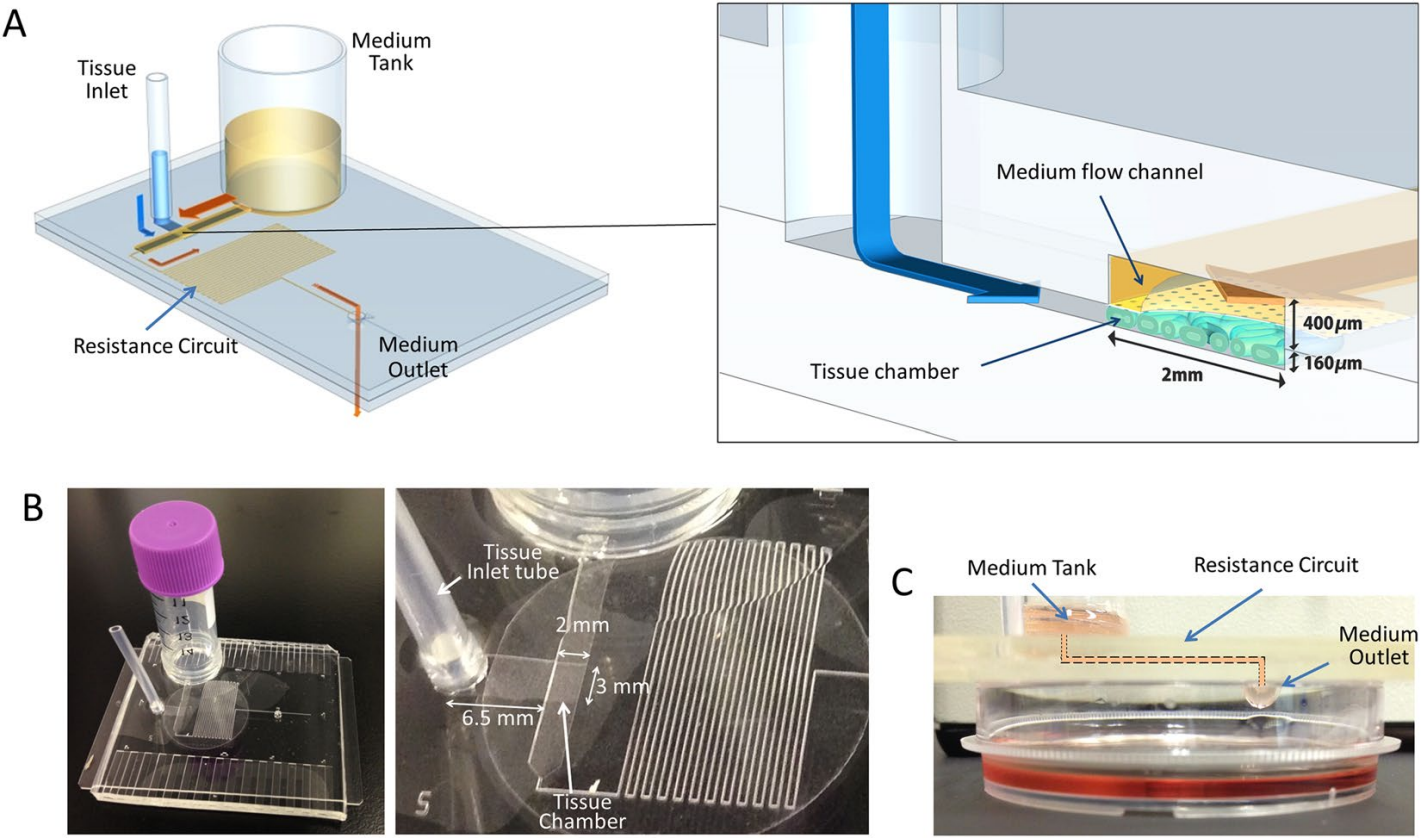
The pumpless microfluidic device. (**A**) Schematic 3-D image of the PL microfluidic device, showing medium tank, tissue inlet, resistance circuit, and medium outlet. On the right, an enlarged view of the portion encompassing the tissue chamber, medium flow channel, and tissue inlet route is shown. (**B**) Photographs of the device. In the right, closer view of tissue chamber and resistance circuit is shown. (**C**) Low-lateral view of the device demonstrating the medium flow route, finally dropping down to the collecting dish.

The PL device was designed based on our previously developed microfluidic (MF) device^3^. The microfluidic platform was composed of two PDMS layers separated by a porous polycarbonate membrane with pores of 10 μm in diameter. The upper layer had a medium flow channel (2 mm in width and 400 μm in height) and a resistance circuit to regulate the medium-flow rate (Fig. 1A). The bottom layer had a sample tissue chamber of 2 mm × 3 mm × 160 µm (W × L × H), a tissue inlet channel of 3 mm × 6.5 mm × 160 µm (W × L × H), and a medium outlet hole. The culture medium in the medium tank passed through the medium flow channel and resistance circuit, and then dropped down out through the outlet (Fig. 1A–C).

**Figure 2:**
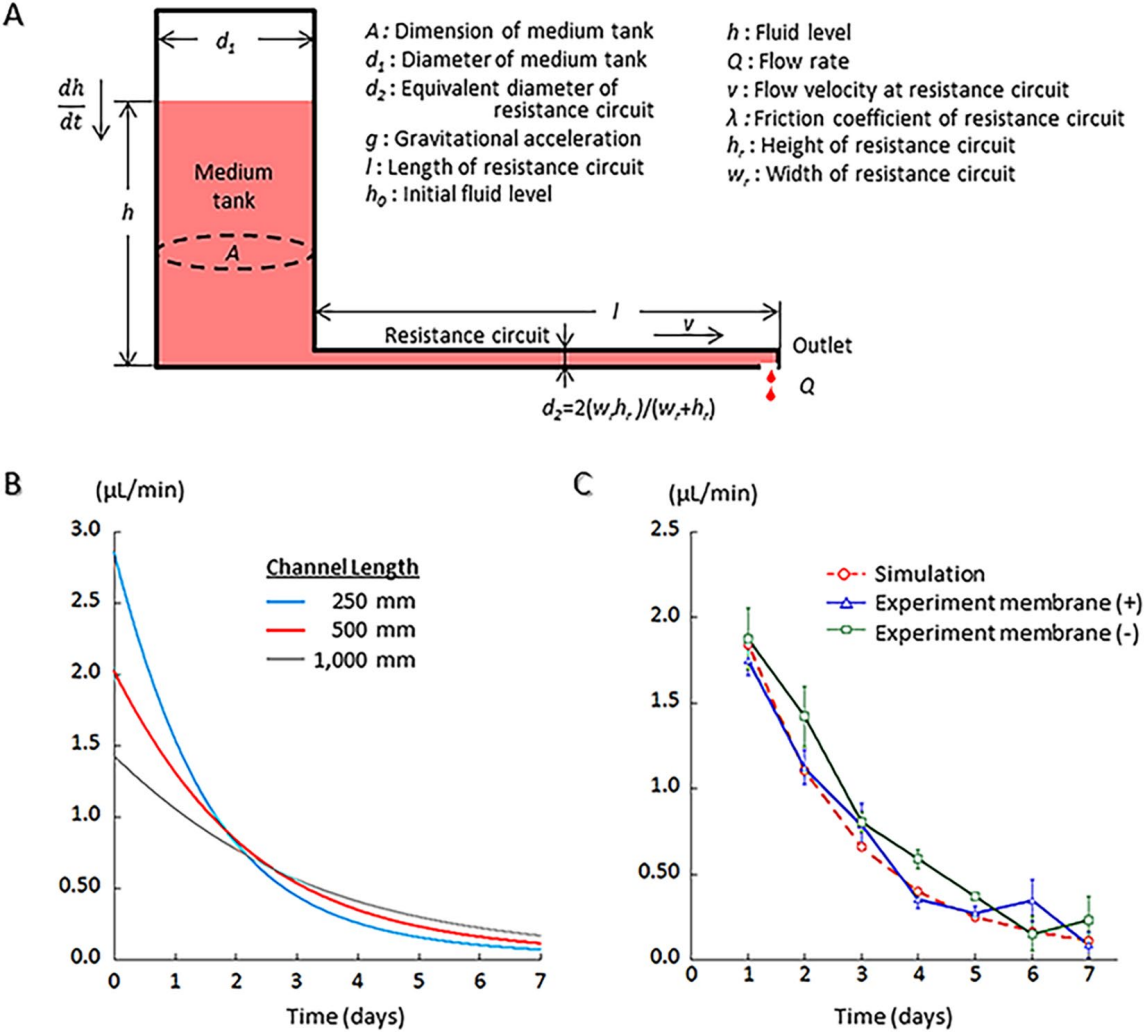
The medium flow simulation. (**A**) Schematic mode of the PL microfluidic device with parameters used for the calculation of flow rate. (**B**) Results of the medium flow simulation. (**C**) Comparison of the results of the simulation with one of the experiments, showing they matched well.

To establish medium perfusion based on the difference in gravitational force between the fluid level in the tank and at the outlet, the flow velocity in the channel was estimated using Bernoulli’s equation:

1 $$h=\frac{{v}^{2}}{2g}+\lambda \frac{l}{{d}_{2}}\frac{{v}^{2}}{2g}$$

where *h* is the fluid level in the tank, *v* is the flow velocity at the resistance circuit, *l* is the length of the resistance circuit, *d*_2_ is the equivalent diameter of the resistance circuit, *g* is gravitational acceleration, and *λ* is the coefficient of channel friction that can be theoretically described as *λ* = 64/*Re*, where *Re* is Reynold’s number. Moreover, *dh/dt* can be described as follows:

2 $$\frac{{dh}}{{dt}}=\frac{Q}{A}=\frac{{{d}_{2}}^{2}}{{{d}_{1}}^{2}}v$$

where *Q* is the flow rate at the outlet, and *A* and *d*_1_ are the planer dimension and diameter of the medium tank, respectively. From Eq. (1) and (2), *v* and *h* can be calculated. Therefore, both the flow velocity and flow rate are adjustable through the alteration of the length and equivalent diameter of the resistance circuit. Flow resistance of the medium flow channel is negligible because the resistance is markedly lower than that of the resistance circuit (Fig. 2A).

Our previous study demonstrated that, in order to maintain *in vitro* spermatogenesis, a flow rate of more than 0.05 µL/min would be required. Therefore, the size of the resistance circuit in the PL device was adjusted to keep the rate of medium flow above that for one week. First, the width (*w*_*r*_) and height (*h*_*r*_) of the resistance circuit were set at 100 and 400 µm, respectively, to be the same as in our previous pump-driven device^3^. Then, different lengths of the resistance circuit, 250, 500, and 1,000 mm, were applied for estimation of the flow rate using the above formulas. As the length extends, the flow rate becomes steadier and can be maintained for longer (Fig. 2B). A flow rate over 0.05 µL/min was maintained over 5 days with lengths of 500 and 1,000 mm (Fig. 2B). We chose 500 mm to fabricate a device and performed an experiment to measure the residual volume of medium in the tank to calculate the flow rate. The experiment was performed with two different devices: one with and the other without a porous membrane inserted between two layers of PDMS, considering that the membrane-insertion might give additional resistance to the flow. Results of this flow rate experiment, performed using 6 devices, 3 with and 3 without a porous membrane, matched well with the simulation, regardless of porous membrane presence or absence (Fig. 2C).

### The induction and maintenance of spermatogenesis in PL device

The GFP expression pattern in the testis of *Acr-Gfp* transgenic mice reliably reflects the progression of spermatogenesis^22–24^. The cytoplasmic GFP appears when a meiotic germ cell reaches the mid-pachytene stage. The GFP then aggregates to form a dot-like structure in the early round spermatid stage, followed by changing into a cap-like form in the late round spermatid stage, and this cap-like form is progressively skewed during the elongating spermatid stage^22^. The spread of GFP-positive cells in the seminiferous tubules also correlates well with the occurrence and progression of spermatogenesis^1–3^. Using this GFP expression, we monitored the spermatogenesis in the cultured tissues.

**Figure 3:**
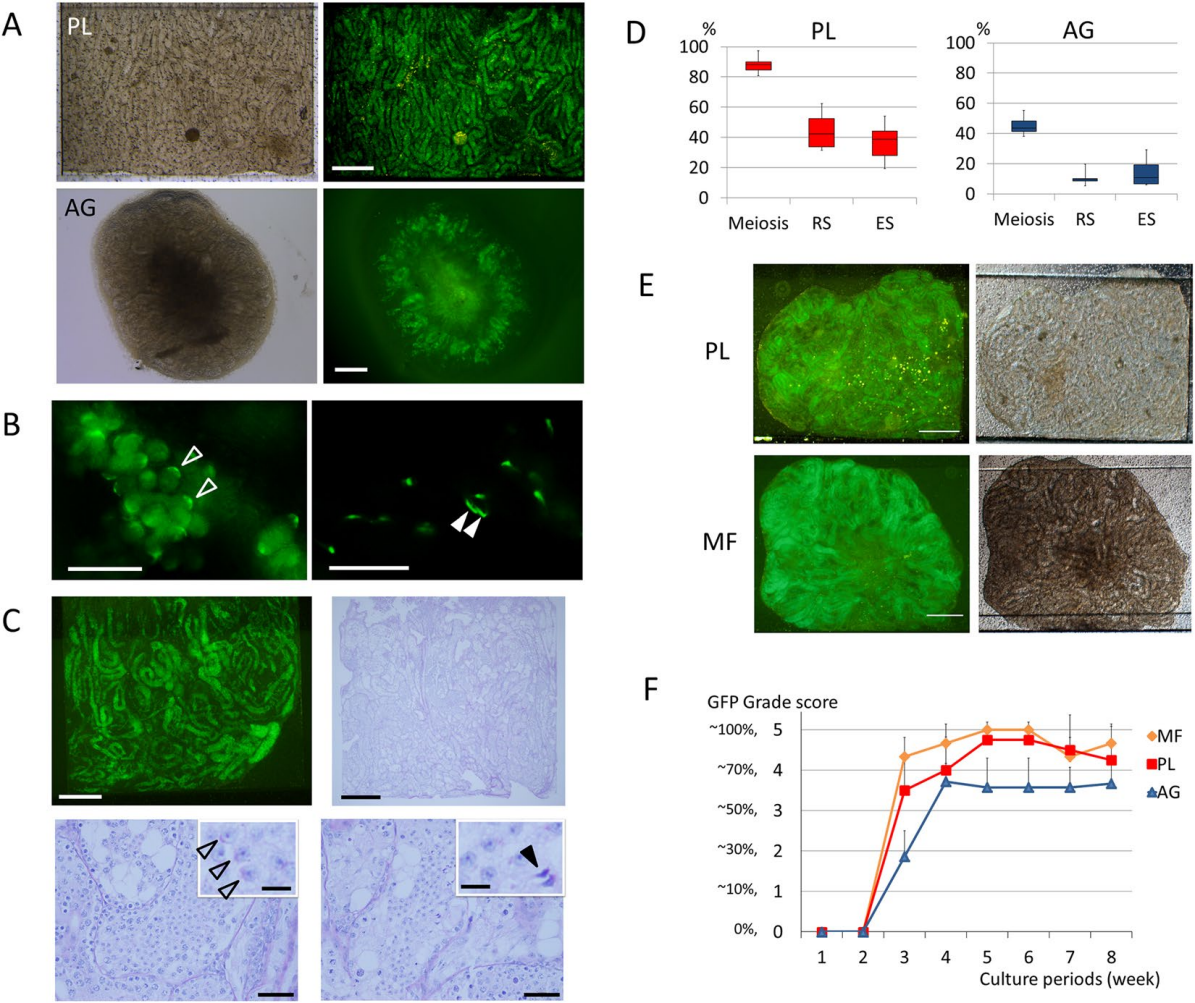
Spermatogenesis in PL device. (**A**) A testis tissue fragment of an *Acr-Gfp* mouse, at 4.5 dpp, was spread flat in MF, and expressed GFP uniformly, pictured at 41 days in culture. The central area of the tissue cultured with the AG method appeared greenish but lacked GFP expression. (**B**) In the tissue of the 3.5 dpp transgenic mouse, cultured for 39 days, cap-like and arched-shaped GFP condensations were observed, indicating the development of round (blank arrowhead) and elongating (solid arrowhead) spermatids, respectively. (**C**) Testis tissue cultured for 66 days maintained uniform GFP expression. In the same tissue, histological examination showed many germ cells including spermatogonia, spermatocytes, round spermatids (blank arrowhead), and elongating (solid arrowhead) spermatids filling seminiferous tubules. (**D**) The rates of the seminiferous tubules containing meiotic germ cells (meiosis), round spermatids (RS), and elongating spermatids (ES) in the 7th week of culture were compared in PL (red) and AG (blue). (**E**) *Acr-Gfp* mouse, 0.5 dpp, testis tissues were cultured in PL and MF devices for 54 days. Extensive GFP expression throughout the tissue was observed in both devices. (**F**) The proportion of GFP expression area was monitored for 8 weeks in each group of MF, PL, and AG. Scale bars: 500 µm (**A**,**E**), 25 µm (**B**), 500 µm (**C**, upper), 50 µm (**C**, lower), and 10 µm (**C**, inlet).

The testis tissue of a neonatal *Acr-Gfp* mouse was spread flat in the tissue chamber of the PL device. When the tissue had been appropriately loaded, the GFP expression started mostly at the same timing as it does in the body at the age of 16~17 days post-partum (dpp). For example, when 4.5 dpp mouse testis tissue was cultured, GFP expression was observed on culture day 12~13 onward. The spatial pattern of the GFP expression was evenly distributed throughout each tissue, being contrary to that cultured on agarose gel (AG) whereby the central region mostly lacks GFP expression, likely due to shortages of oxygen and nutrition (Fig. 3A). On culturing for 39 days, under observation with a higher magnification, round and elongating spermatids were recognized as condensing GFP shaping cap-like or arched structures, indicating the production of round and elongating spermatids, respectively (Fig. 3B). Histological examination also confirmed the progression of spermatogenesis in the PL device. Testis tissue cultured for 66 days, expressing GFP extensively, was fixed and stained with Periodic Acid–Schiff (PAS) stain, by which the acrosomal structure, a hallmark of haploid cells, was stained violet. Meiotic germ cells were observed in most seminiferous tubules. Haploid cells including round spermatids and elongating spermatids were also confirmed by the PAS stain (Fig. 3C). The rates of tubules containing meiotic germ cells, round, and elongating spermatids were 85, 44, and 28%, respectively, in the PL device (tissue number = 5), while they were 43, 5, and 2% in those cultured on AG (tissue number = 6) (Fig. 3D). Then, we performed culture experiments to compare PL and MF devices along with the AG method. Three experiments were performed using *Acr-Gfp* mouse testes at the age of 0.5 to 3.5 dpp; 4 samples in the PL device, 5 samples in the MF device and 7 samples in the AG method in total. They were cultured for 56 days and the extent of the GFP expression was monitored. Both PL and MF induced and maintained the same level of GFP expression whereby more than 70% of the area of both tissues comprised a GFP-positive area, meaning that the induction of spermatogenesis up to or beyond the pachytene meiotic phase was taking place in a broad region (Fig. 3E,F). These GFP-positive areas were maintained in both PL and MF until the end of the experiment, while tissues in AG showed smaller GFP-positive, 50 to 60%, areas (Fig. 3F). These results demonstrated that the PL device is as effective as MF in inducing and maintaining mouse spermatogenesis.

### The maintenance of spermatogonia in PL device

**Figure 4:**
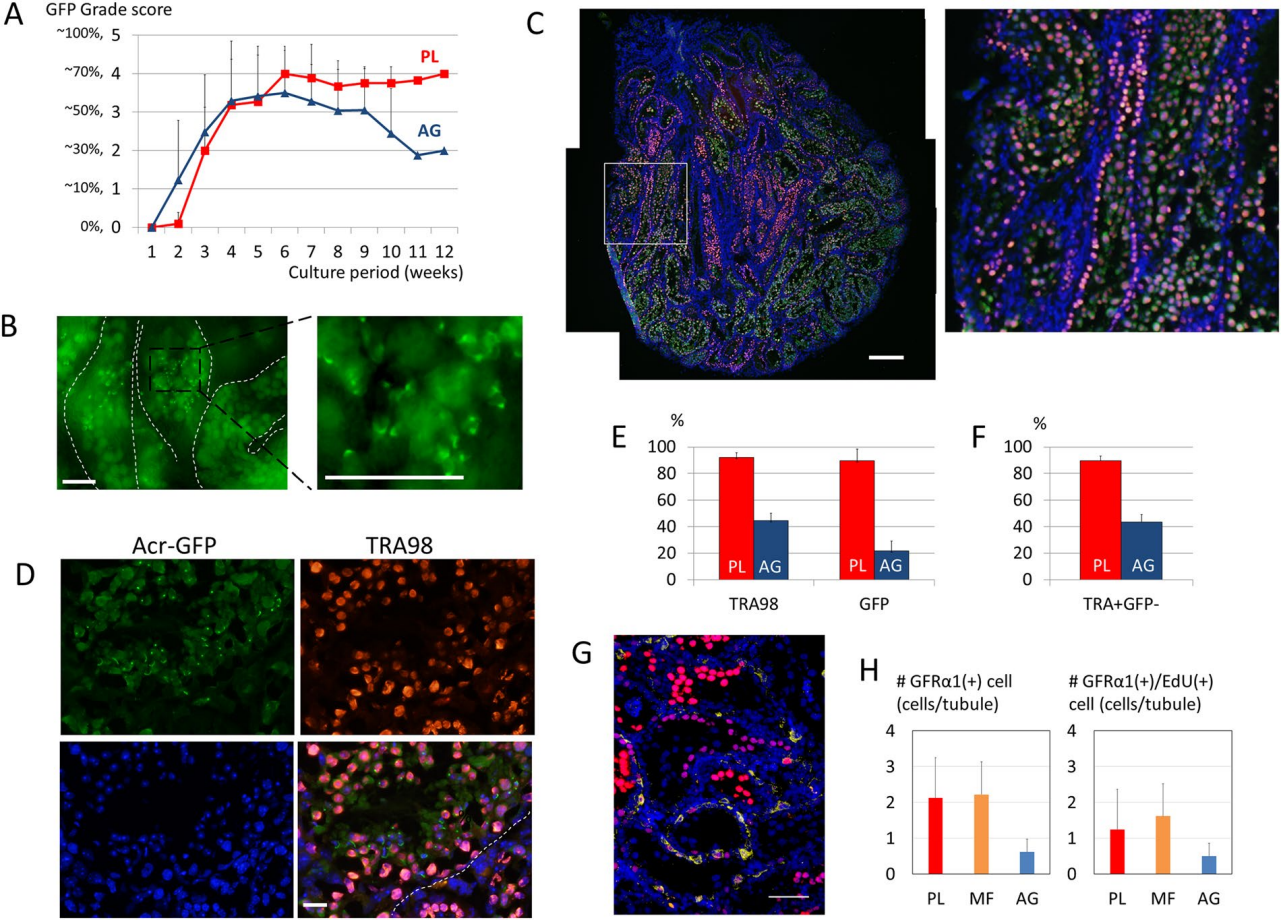
The maintenance of spermatogonia in PL device. (**A**) The average GFP-grade transition was compared between PL (red line) and AG (blue line) for 12 weeks. (**B**) At 104 culture days, the seminiferous tubules of the 2.5 dpp transgenic mouse contained GFP-positive cells including many haploid cells. (**C**) Immunohistological examination of the tissue cultured for 89 days at a lower magnification demonstrated GFP(+) (green) and TRA98(+)cells (red), counter-stained with Hoechst (blue). The white rectangular area is enlarged on the right. (**D**) Immunohistological examination at a higher magnification, GFP(+)/TRA98(+) cells were located in the inner area of the seminiferous tubule, while GFP(−)/TRA98(+) cells were near the basal membrane (broken line). Dot and crescent-like GFP condensations observed in the inner area of the seminiferous tubule represent round and elongating spermatids, respectively. (Upper left panel) (**E**) The rates of seminiferous tubules containing TRA98(+) and GFP(+) cells, respectively, at 12 culture weeks were compared between PL (red) and AG (blue). (**F**) The rates of the seminiferous tubules containing TRA98(+)/GFP(−) cells in PL (red) and AG (blue). (**G**) Immunohistological examination of the tissue cultured for 54 days. GFRα1(+) cells are in yellow and EdU(+) cells are in red. The nuclei were stained by Hoechst 33342 dye (blue). (**H**) The number of the GFRα1(+) cells and GFRα1(+)/EdU(+) cells per seminiferous tubule at 8 culture weeks were compared between PL (red), MF (orange), and AG (blue). Scale bar: 50 µm (**B**), 200 µm (**C**), 20 µm (**D**), 50 µm (**G**).

As in the case of the MF device, the PL device was able to maintain spermatogenesis for longer than the AG method. In fact, in the following 7 experiments, which included 11 samples in the PL device and 39 samples in AG culture, using mice aged from 0.5 to 4.5 dpp (average: 2.1 dpp), spermatogenesis in PL was maintained for over 12 weeks in up to a 70% area of the tissue, while tissue on AG gradually lost spermatogenesis at later than 8 weeks, dropping to 30% (Fig. 4A). With such long-lasting spermatogenesis in the PL device, haploid cells were also observed. Forexample, at 104 days of incubation, the testis tissue in the device contained GFP-positive germ cells including haploid cells (Fig. 4B). These observations strongly suggest that spermatogonia including the spermatogonial stem cell population were maintained in the PL as well as MF devices. In order to identify the types of germ cells, we performed immunohistological examination using antibodies to TAR98, a pan-germ cell marker, GFRα1, a marker for undifferentiated spermatogonia, and to EdU and GFP. First, we performed immunostaining against TRA98 and GFP. A testis tissue, age 2.5 dpp, cultured in the PL device for 89 days contained many GFP-positive cells, corresponding to meiotic or post-meiotic germ cells, in a large area of the tissue (Fig. 4C). On closer observation, post-meiotic cells were recognized with cap-like or arched structures of GFP, which identified them as haploid cells (Fig. 4D). At the same time, the majority of seminiferous tubules were filled with TRA98(+)/GFP(−) cells in their basal compartment, at the periphery of the tubules, which represented spermatogonia (Fig. 4D). The rates of seminiferous tubules containing TRA98(+) and GFP(+) cells were 92 and 90%, respectively, in tissues cultured in the PL device. On the other hand, they were 45 and 22% in cases of AG culture, respectively (Fig. 4E). Cells that were TRA98(+)/GFP(−), representing spermatogonia, were recognized in 90% of seminiferous tubules in the PL group, but 44% in the AG group (Fig. 4F). In addition, the EdU treatment at 8 weeks of culture followed by immunostaining against EdU and GFRα1 one week later was performed to identify populations of undifferentiated spermatogonia, which include spermatogonial stem cells, and evaluate their proliferative activity. GFRα1(+) cells were all located adjacent to the basement membrane, the location where they should be, and some of them were positive for EdU, indicating they were proliferating (Fig. 4G). GFRα1(+) cells were counted in the sections of the cultured tissues (n = 3, 3, and 6 in PL, MF, and AG, respectively) to be 2.1, 2.2, and 0.6 cells/tubule in PL, MF, and AG groups, respectively. GFRα1(+)/EdU(+) cells were also counted as 1.2, 1.6, and 0.5 cells/tubule in PL, MF, and AG, respectively (Fig. 4H). Although there was no significant difference among groups, the PL device couldmaintain the spermatogonial population as well as the MF device and possibly better than the AG method.

## Discussion

In our previous study, we succeeded in functionally maintaining mouse testis tissue in a microfluidic device for as long as 6 months. Sperm and round spermatids harvested in this prolonged period were sufficiently functional to produce offspring with micro-insemination. In addition, testosterone production, another important function of the testis, was also maintained in the device^3^. This study therefore clearly demonstrated the effectiveness of the microfluidic system to support the functional integrity of the tissue for longer periods than regular organ culture methods, namely the gas-liquid interphase method. The merits of the MF system over the AG method can be summarized as follows: First, MF allows efficient expression of the function of the cultured tissue. Second, it allows clear and detailed observation. Third, it can maintain the tissue structure and functions for long periods; being less stressful to the tissue. However, such advantages come with expenses. First of all, when introducing the MF system initially in a laboratory, certain investment is necessary. Producing each MF device requires experience and skill, let alone time, labor, and financial expenses. Culturing with the MF device also involves other problems. Introducing a tissue into the device can be troublesome. Changing or adding medium needs careful handling of the device at all times. Space occupancy in an incubator per tissue might be larger than with the AG method. In addition, the sample number is also limited, not by the number of devices, but by the number of external pumps or syringes adjusted to the pump. These inconveniences we encountered in our use of the MF device motivated us to develop a new MF device. Among others, we considered the tube connection between the device and external pump as the most serious constraint of the system, although it is a critical part of the fluidic system. Several pump-free MF systems have been reported^7–19^. However, regarding the organ culture, we found only one relevant report on a study that cultured a human skin-equivalent composed of an extracellular matrix and primary human skin cells^20^. Although pumpless, it used a rocking platform system to make the culture fluid flow in the channel of the device, also rendering the system dependent on an external-power source.

In the present study, we developed a microfluidic device that works without an external power source. We used hydrostatic pressure and a resistance circuit to induce slow and long-lasting medium flow in the channel. The basic design of the device was adopted based on our previous one and we fixed the size of the channel, especially the height, at 400 µm, because changing it might significantly influence the culture results. Then, we simulated the design of the resistance circuit and found that a 100-µm width and 500-mm length, along with a 400-µm height, will maintain the flow rate in the expected range. The actual experiment confirmed that the simulation was accurate. Our PL device, as a result, demonstrated the ability to culture mouse testis tissue and it induced spermatogenesis as efficiently as our previous device for over 3 months. Through this experiment with the PL device, we recognized that changes in the medium flow rate, at least between 0.05 and around 2.0 µL/min, did not affect the performance of testis tissue culture in practice. These results can be expected, because the device, with the porous membrane between the flowing medium and the tissue, delivers medium contents to cells through diffusion-basis. Thus, the exact velocity of flowing medium *per se* does not matter, as long as it is above a certain threshold. When more accurate control of flow rate is required in certain types of experiment, however, the design of the device could be modified, such as by using a reservoir tank with a larger diameter or introducing a horizontally orientated reservoir^7,15,21^.

This is the first report, to our best knowledge, of a pumpless MF device that maintained tissue and its function for such a long period. In order to improve the device, there are several important points to consider. One of them is reducing the size of the device, especially by minimizing the resistance circuit. This may be possible by narrowing its caliber or reducing the height, which would produce a more constant flow rate in our simulation. Such a remodeling of the MF device along with other improvements would contribute not only to testis tissue culture but also to organ culture in general.

## Materials and Methods

### Device fabrication

The microfluidic device using PDMS as a material was produced by conventional photolithography and soft lithography techniques^25–27^, as in our previous report^3^. Briefly, the SU-8 mold master was initially made, serving as a mold for the production of PDMS layers. Holes in the PDMS layers for medium inlet and outlet and tissue-inlet were made using a dermapunch. The two layers and thin porous polycarbonate membrane in between were bonded together using the common O_2_ plasma method. Silicone tubes (3 mm outer diameter and 2 mm inner diameter) for the tissue inlet and a medium reservoir tank, which was the cut half of a polypropylene tube (Falcon™ 15 mL Conical Centrifuge Tubes, Corning, USA), were fixed by PDMS. Devices were sterilized by UV irradiation before use.

### Simulation and measurement of medium flow rate in PL device

Fluid flow rate simulation was performed based on equations described in Results, using Mathematica (Wolfram, USA). The width and height of the resistance circuit channel were set at 100 and 400 µm, respectively. The value of *h*_*0*_, which is the initial fluid level in the medium tank, was set at 40 mm, the same as in the culture experiment. The values of *A*, *d*_*1*_, and *g* were defined as 176.715 mm^2^, 15 mm, and 9.80665 m/s^2^, respectively. The value of *d*_2_ was calculated using the equation *d*_2_ *= *2(*w*_*r*_ *h*_*r*_)*/(w*_*r*_+*h*_*r*_ *)*, where *w*_*rc*_ and *h*_*rc*_ are the width and height of the resistance circuit channel, respectively. Then, different lengths of the circuit were set to run the simulation. The accuracy of the simulation data was tested with devices by measuring the actual flow rate of fluid of the culture media.

### Animals

*Acr-Gfp* transgenic mice^22,23^ provided by RIKEN BRC through the National Bio-Resource Project of MEXT, Japan were used as the testis tissue source. For mating, males homozygous for *Acr-Gfp* were used as sires, while females were either homozygous, heterozygous, or the wild-type. B6D2F1 female mice (Japan SLC, Inc.) and ICR female mice (CLEA Japan, Inc.) were used for oocyte collection and embryo transfer, respectively. Mice were housed in air-conditioned rooms at 24 ± 1 °C and 55 ± 5%, with a 14-hour-light and 10-hour-dark lighting cycle. The mice were kept in a specific pathogen-free room. Commercially made hard pellets (MF, Oriental Yeast, Japan) were fed ad libitum. Drinking water was acidified to pH 2.8–3.0 using HCl. All animal experiments conformed to the Guide for the Care and Use of Laboratory Animals and were approved by the Institutional Committees of Laboratory Animal Experimentation (Animal Research Center of Yokohama City University, Yokohama, Japan, and RIKEN Tsukuba Institute, Japan).

### Culture of testis tissues

Testes of neonatal mice at 0.5~4.5 days postpartum (dpp) were decapsulated by forceps and the tissue was torn with the forceps, if necessary, into about 1 mm^3^. They were randomly allocated to the PL, MF, or AG group for culturing in PL, MF, or on agarose gels, respectively. As for the PL and MF methods, tissue was pushed into the tissue chamber through the tissue inlets by a micropipette. Specifically, the tissue put into the inlet tube was allowed to sink down to the bottom. Then, the device was tilted to make the tissue chamber lower than the bottom of the inlet tube. A micropipette was used to apply some pressure to push the tissue toward the chamber to settle it. The culture medium was loaded in the medium tank twice a week (approximately 3.0 mL/tank) to initialize the fluid level of the tank at 40 mm. Tissues of the AG group were cultured on agarose stands (1.5% w/v) placed in wells of a 12-well culture plate (CELLSTAR® Tissue Culture Plates, Greiner Bio-One)^1,2,28^. Each gel was loaded with 1–3 testis tissue fragments. The amount of medium was adjusted so that it would come up to half-to-four-fifths of the height of the agarose gel (approximately 0.5 mL/well). Medium change was performed once a week. The culture incubator was supplied with 5% CO_2_ in air and maintained at 34 °C. The culture medium used for organ culture was α-Minimum essential medium (α-MEM) (Invitrogen: 12000-022), supplemented with 40 mg/mL AlbuMAX (Invitrogen: 10828-028). In the 5-ethynyl-2′-deoxyuridine (EdU) labelling procedure, the testis tissue cultured for about 50 days EdU (Invitrogen: C10338) was added to the medium (5-µM final concentration) at around 50 days of culture for 21 hours. Then, the testis tissue was cultured in the culture medium without EdU for one week and fixed for the detection of EdU.

### Gross and histological examination

Tissues in culture were observed every 7 days under a stereomicroscope (Leica M 205 FA; Leica, Germany) to evaluate the efficiency of spermatogenesis by the GFP-expression grade. The area showing GFP expression was estimated visually and classified into one of six grades: G0 to G5, based on our previous GFP-expression grading scale^1,2,28^. Briefly, G0 to G5 corresponds to 0, ~10, ~30, ~50, ~70, and ~100% of the area of GFP expression, respectively, on observation with stereomicroscopy. An inverted microscope (Olympus IX 73; Olympus, Tokyo, Japan) was used to observe the GFP-expressing germ cells more closely to distinguish steps of spermatids. For histological examination, the specimens were fixed with Bouin’s fixative and embedded in paraffin. One section showing the largest cut surface was made for each specimen and stained with Periodic acid-Schiff (PAS).

### Immunohistochemical examination

For immunofluorescence staining, tissues fixed with 4% paraformaldehyde in PBS were cryo-embedded in OCT compound (Sakura Finetechnical, Japan) and cut into 7-µm-thick sections. Nuclei were counterstained with Hoechst 33342 dye. Specimens were observed with a microscope (Olympus IX 73; Olympus, Tokyo, Japan). The following were used as primary antibodies: chicken anti-GFP antibody (1:1,000, Nakalai Tesque, Inc., Kyoto, Japan), rat anti-TRA98 antibody (1:200, Abcam, USA), and anti-GFRα1 antibody (1:200, R&D Systems, USA). The secondary antibodies used were mouse anti-chicken IgG and goat anti-rat IgG, conjugated with Alexa 488 or Alexa 555 (1:200; Life Technologies, USA), and donkey anti-goat IgG conjugated with Alexa 647 (1:200; Life Technologies, USA). For the detection of EdU, sections were treated with the Click-iTTM EdU Alexa FluorTM 555 Imaging Kit (Invitrogen: C10338) before exposure to the primary and secondary antibodies on immunohistochemical examination.
